# Supplementary material for: Content-rich biological network constructed by mining PubMed abstracts
Source: BMC Bioinformatics. 2004 Oct 8;5:147. doi: 10.1186/1471-2105-5-147 (PMC528731; doi:10.1186/1471-2105-5-147)
Supplement: Additional File 2 — The original results of the above study (non-essential files are deleted to keep the file size under the limit set by BMC bioinformatics). [file 1471-2105-5-147-S2.bz2 › chilibotAdditionalFile2/dip40/47ID7824954E188/html/SP1_GTF2B.html]

 


 **SP1** and **GTF2B** 
  
Found 32 abstracts in PubMed, retrieved 32.  
 

 What does Google say? 
 PDF only 
| .edu only 

---

**Interactive relationship** (e.g. stimulation, inhibition, etc)

**Neutral relationship**- Mutational analyses demonstrate that the CCCGCG sequence in footprint IIb  [ **GTF2B** ]  is critical for  **Sp1**  binding and P2 promoter activity.  Ref: 9287317 J Biol Chem, 1997
- An Ets  **Sp1**  interaction in the 5 flanking region of the megakaryocyte specific alpha IIb  [ **GTF2B** ]  gene appears to stabilize  **Sp1**  binding and is essential for expression of this TATA less gene.  Ref: 8822926 Blood, 1996
- The fact that YY1 interacts with a number of key regulatory proteins e.g. TBP, TFIIB  [ **GTF2B** ] , TAFII55,  **Sp1** , and E1A has suggested that these interactions are important for determining which particular function of YY1 is displayed at a specific promoter.  Ref: 10452940 Gene, 1999
- Here we show that, in DDT1MF 2 smooth muscle cells, the major protein bound to footprint II is not NF1 but  **Sp1** , which binds to the 5 portion of the footprint II sequence footprint IIb  [ **GTF2B** ] .  Ref: 9287317 J Biol Chem, 1997
- We also show that  **Sp1**  bound to the promoter is essential for the DNA looping and recruitment of basal transcription factors such as TFIIB  [ **GTF2B** ]  and Cdk7 but not for recruitment of TBP.  Ref: 10468567 Proc Natl Acad Sci U S A, 1999
- Repression assays revealed that WT1 represses all three classes of activation domains  **Sp1**  and CTF, which stimulate initiation type I, human immunodeficiency virus type I Tat fused to a DNA binding domain, which stimulates predominantly elongation type IIA, and VP16, p53 and E2F1, which stimulate both initiation and elongation type IIB  [ **GTF2B** ] .  Ref: 10390530 Nucleic Acids Res, 1999

**Non-interactive relationship** (e.g. studied together, co-existance, homology, etc.)

- When the general transcription factors GTFs were added in the assay, NC enhances NF kappaB,  **Sp1** , and TFIIB  [ **GTF2B** ]  induced HIV 1 LTR directed RNA transcription.  Ref: 10704334 Virology, 2000
- We demonstrate that the recruitment of promoter proximal activator  **Sp1** , and the components of the basal transcription factors such as TBP, TFIIB  [ **GTF2B** ] , and Cdk7, is enhanced with thyroid hormone activation.  Ref: 10468567 Proc Natl Acad Sci U S A, 1999
- Our results suggested that group IIb  [ **GTF2B** ]  metals Zn II, Cd II, and Hg II were able to complex with the peptide and bind the double stranded DNA with high affinity as well as inhibiting  **Sp1**  DNA binding activity in a concentration dependent manner.  Ref: 10873713 Toxicol Appl Pharmacol, 2000
- Chromatin immunoprecipitation revealed binding of NF Y,  **Sp1**  and TFIIB  [ **GTF2B** ]  to the promoter in vivo.  Ref: 12586348 FEBS Lett, 2003
- We show here that i the Tag domain containing amino acids 133 to 249 directly interacts with the general transcription factor TFIIB  [ **GTF2B** ] , the activator protein  **Sp1** , and the 140 kDa subunit of RNA polymerase II, as well as with TBP and TEF 1.  Ref: 8551580 J Virol, 1996
- Here, we show that the p50 subunit as well as the p50 p65 of NF kappaB, and not other factors such as  **SP1** , TFIIB  [ **GTF2B** ] , polymerase II, TFIIA, or p65, can be acetylated by CBP p300 HAT domain.  Ref: 11739381 J Biol Chem, 2002
- In the present study, we used general transcription factors from HeLa cell nuclear extracts, recombinant TATA binding protein TBP and TFIIB  [ **GTF2B** ] , and the transcriptional activator  **Sp1**  to reconstitute in vitro transcription for the ICP4 promoter and to examine the effects of purified ICP4 on transcription.  Ref: 8415735 Proc Natl Acad Sci U S A, 1993
- Mutations have suggested that the N terminal domain of Vpr encompassing the first 40 residues could be required for nuclear localization, packaging into virions and binding of transcription factor TFIIB  [ **GTF2B** ] ,  **Sp1** , viral proteins p6 and cellular proteins RIP1, UNG, karyopherins.  Ref: 10561576 Eur J Biochem, 1999
